# The Growth of *Steroidobacter agariperforans* sp. nov., a Novel Agar-Degrading Bacterium Isolated from Soil, is Enhanced by the Diffusible Metabolites Produced by Bacteria Belonging to *Rhizobiales*

**DOI:** 10.1264/jsme2.ME13169

**Published:** 2014-03-13

**Authors:** Masao Sakai, Akifumi Hosoda, Kenjiro Ogura, Makoto Ikenaga

**Affiliations:** 1 Faculty of Agriculture, Kagoshima University, 1–21–24, Korimoto, Kagoshima 890–0065, Japan; 2 School of Agriculture, Meijo University, 1–501 Shiogamaguchi, Tenpaku-ku, Nagoya, Aichi 468–8502, Japan; 3 Graduate School of Bioresource and Bioenvironmental Sciences, Kyushu University, 6–10–1, Hakozaki, Higashi-ku, Fukuoka 812–8581, Japan

**Keywords:** agar-degrading bacterium, *Gammaproteobacteria*, novel species, *Steroidobacter*

## Abstract

An agar-degrading bacterium was isolated from soil collected in a vegetable cropping field. The growth of this isolate was enhanced by supplying culture supernatants of bacteria belonging to the order *Rhizobiales*. Phylogenetic analysis based on 16S rRNA gene sequences indicated the novel bacterium, strain KA5–B^T^, belonged to the genus *Steroidobacter* in *Gammaproteobacteria*, but differed from its closest relative, *Steroidobacter denitrificans* FS^T^, at the species level with 96.5% similarity. Strain KA5–B^T^ was strictly aerobic, Gram-negative, non-motile, non-spore forming, and had a straight to slightly curved rod shape. Cytochrome oxidase and catalase activities were positive. The strain grew on media containing culture supernatants in a temperature range of 15–37°C and between pH 4.5 and 9.0, with optimal growth occurring at 30°C and pH 6.0–8.0. No growth occurred at 10 or 42°C or at NaCl concentrations more than 3% (w/v). The main cellular fatty acids were iso–C_15:0_, C_16:1_ω7c, and iso–C_17:1_ω9c. The main quinone was ubiquinone-8 and DNA G+C content was 62.9 mol%. In contrast, strain FS^T^ was motile, did not grow on the agar plate, and its dominant cellular fatty acids were C_15:0_ and C_17:1_ω8c. Based on its phylogenetic and phenotypic properties, strain KA5–B^T^ (JCM 18477^T^ = KCTC 32107^T^) represents a novel species in genus *Steroidobacter*, for which the name *Steroidobacter agariperforans* sp. nov. is proposed.

Several types of agarase-producing bacteria that degrade and utilize agar have been isolated to date. The most recently reported agar-degrading bacteria have generally been isolated from marine environments (4, 25, 28). Agar is a polysaccharide produced by marine red algae; therefore, it is reasonable to assume that most agar-degrading bacteria inhabit marine ecosystems. On the other hand, several agar-degrading bacteria have previously been isolated from non-marine environments, including *Cytophaga* sp. and *Alteromonas* sp., which were isolated from freshwater sources (1), *Spirochaeta alkalica*, from soda lakes (46), and *Bacillus* sp., *Cytophaga* sp., and *Streptomyces coelicolor*, from soil (17, 35, 43). We also recently reported the existence of agar-degrading bacteria in plant rhizospheres (15, 16). Mucilage polysaccharides (the structure of which are unknown) secreted from plant roots have been shown to contain abundant amounts of galactose as their main sugar component (3, 5, 21, 26), which suggests that agar-like polysaccharides may be present in plant rhizospheres. The agardegrading enzymes of these isolates may degrade mucilage polysaccharides. Previous studies on agar-degrading bacteria have provided an insight into the ecology of non-marine agar-degrading bacteria.

We isolated a novel agar-degrading bacterium from the soil of a vegetable cropping field. The agarolytic isolate, designated strain KA5–B^T^, did not grow well on any media, but exhibited a commensal interaction with a bacterium belonging to the order *Rhizobiales*. Consequently, strain KA5–B^T^ was successfully isolated using a medium containing culture supernatant of this bacterium. The 16S rRNA gene sequence of strain KA5–B^T^ was classified into genus *Steroidobacter* in *Gammaproteobacteria*. However, it was significantly differentiated from its closest relative, *Steroidobacter denitrificans* FS^T^ (8), which is the sole species within this genus, at the species level. The aim of the present study was to investigate the commensal interaction between strain KA5–B^T^ and its companions and determine the taxonomic status of this strain in the genus *Steroidobacter* using a polyphasic approach. Strain KA–5B^T^ formed a novel species, for which the name *Steroidobacter agariperforans* sp. nov. is proposed.

## Materials and Methods

### Soil collection and cultivation after enriching agar-degrading bacteria

An agar-degrading bacterium, strain KA5–B^T^, was isolated from agricultural soil using the following procedures. A soil sample was collected from a vegetable cropping field located in Fukuoka prefecture, Japan. Agar was dissolved in distilled water and sterilized. The soil sample was added to the agar to a final concentration of 1.0% (w/w, agar/dry soil). The water content was adjusted to 50% (w/w, water/dry soil). Agar–supplemented soil was packed in bottles and incubated at 28°C for 14 d to enrich agar-degrading bacteria. The soil samples were then suspended in 10 mM phosphate buffer solution (pH 7.0) and serially diluted. One hundred μL of the diluted portions were spread on PSG medium (1.7 g L^−1^ peptone, 0.3 g L^−1^ soytone, 0.25 g L^−1^ glucose, 0.25 g L^−1^ K_2_HPO_4_, and 0.5 g L^−1^ NaCl) solidified with 1.5% agar. After being incubated at 30°C for 10 d, agar-degrading bacteria in colonies with depressed circumferences were selected and streaking was repeated on PSG agar to isolate pure colonies.

### Culture conditions and isolation of agar-degrading bacteria

Two different morphologies were still observed by microscopy after streaking was repeating and the selected colonies were incubated in PSG liquid. These cells were designated strain KA5–A and strain KA5–B^T^. Streaking on plates and serial dilutions (1:10) failed to isolate the agar-degrading bacterium, and only strain KA5–A formed pure colonies on the PSG agar; however, it had no agar-degrading ability. In contrast to KA5–A, no pure colonies of strain KA5–B^T^ were detected on the agar plates. However, pure colonies of strain KA5–B^T^ were detected on the agar plates after a 5-d incubation at 30°C when supernatants from KA5–A cultures were supplemented into the PSG medium, and these formations had depressed circumferences. Since the growth of strain KA5–B^T^ was extremely poor, and a faint colony only formed on plates inoculated with a high density of the cells. The cells of strain KA5–B^T^, which were cultured at 30°C for 5 d with PSGS medium, solidified with agar containing 10% of the supernatant of the KA5–A culture by PSG liquid or cultured in PSGS liquid at 30°C until the beginning of the stationary phase, were used in the following analyses unless otherwise stated. When it was desirable to avoid the formation of a depressed circumference on the PSGS agar, strain KA5–B^T^ was grown on media solidified with 1.5% (w/v) gellan gum.

### Sequencing of the 16S rRNA gene and phylogenetic analysis

The 16S rRNA gene sequencing of strains KA5–A and KA5–B^T^ was conducted by PCR using two oligonucleotide primers, 5′–AGAGTTTGATCCTGGCTCAG–3′ and 5′–AAGGAGGTGATCC AGCC–3′ (corresponding to positions 8–27 and 1525–1541 of the *Escherichia coli* 16S rRNA gene) (45). The genomic DNA of these strains was extracted according to the method described by Pitcher *et al.* (30). The amplified 16S rRNA gene was purified with a Qiaquick PCR purification kit (Qiagen) according to the manufacturer’s instructions. The 16S rRNA genes were sequenced directly with a DNA Sequencer using flanking and internal primers. The resultant sequence was used for an initial homology search against sequences in GeneBank using the BLAST program.

Multiple alignments of the KA5–B^T^ sequences were performed with its closest relatives using CLUSTAL W software (41). Phylogenetic trees were constructed by three different methods; neighbor-joining (32), maximum-likelihood (9), and maximum-parsimony (11) algorithms using MEGA software version 5.1 (38) for all analyses. To evaluate the topology of the resultant tree, bootstrap analysis (10) was based on 1,000 resamplings.

### Examination of the commensal interaction that enhanced growth

Seventeen bacteria including strain KA5–A were employed to examine the commensal interaction that enhanced the growth of strain KA5–B^T^. The remaining sixteen bacteria were *Arthrobacter nicotianae* NBRC 14234 and *Streptomyces albus* NBRC 13014 for *Actinobacteria*, *Bacillus subtilis* NBRC 13719 for *Firmicutes*, *Azorhizobium caulinodans* NBRC 14845, *Bradyrhizobium japonicum* USDA 110, *Brevundimonas subvibrioides* NBRC 16000, *Mesorhizobium loti* MAFF 301724, *Rhizobium leguminosarum* NBRC 14778, *Rhizobium meliloti* MAFF 303039, *Rhizobium radiobacter* NBRC 15193, *Rhizobium rhizogenes* MAFF 301724, *Sinorhizobium fredii* IFO 14780 and *Sphingomonas pruni* NBRC 15498 for *Alphaproteobacteria*, *Ralstonia solanacearum* MAFF 730101 for *Beta–proteobacteria*, and *Escherichia coli* DH5α and *Pseudomonas putida* NBRC 14614 for *Gammaproteobacteria*. The respective bacteria were cultured in LB liquid (10 g L^−1^ peptone, 5 g L^−1^ yeast extract, and 5 g L^−1^ NaCl; pH 7.2) at 30°C for 5 d while shaking at 100 rpm with the multi shaker MMS–200 (Tokyo Rikakikai, Tokyo, Japan). After the incubation, the supernatants of cultured bacteria were recovered by centrifuging at 8,000×*g* and 4°C to avoid the collection of cell pellets. They were passed through a membrane filter with 0.22 μm pores to remove residual cells. Filter papers cut to 0.5 cm × 2.5 cm in size were soaked in the cell–free supernatants and placed on the PSG gellan gum plate to prepare two lines in parallel. Strain KA5–B^T^ was subsequently inoculated by streaking side to side between the papers and incubated at 30°C for 5 d. The plates with filter papers soaked in the LB liquid and without filter papers were also prepared as controls.

### Examination of polysaccharide-degrading ability

The degradation ability of strain KA5–B^T^ was investigated using various polysaccharides. Arabinan, wheat arabinoxylan, galactomannan, xyloglucan, debranched arabinan, pectic galactan, larch arabinogalactan, and polygalacturonic acid (1.0 g L^−1^ each), agar, starch, and dextrin (2.0 g L^−1^ each), and carboxymethyl cellulose (CM–cellulose) and xylan (5.0 g L^−1^ each) were individually dissolved in HMM medium (0.125 g L^−1^ Na_2_HPO_4_·2H_2_O, 0.25 g L^−1^ Na_2_SO_4_, 0.32 g L^−1^ NH_4_Cl, 0.18 g L^−1^ MgSO_4_, 0.0067 g L^−1^ FeCl_3_·6H_2_O, 0.013 g L^−1^ CaCl_2_·2H_2_O) solidified with 1.5% gellan gum. The cells of the strain cultured in liquid media were washed with 10 mM sodium phosphate buffer (pH 7.0) after collection by centrifugation and again suspended in sodium phosphate buffer.

Regarding the setup of the experiment, filter paper was soaked in the cell-free supernatants of strain KA5–A prepared using the same procedure as that described in the section on the commensal interaction and was then placed on the HMM gellan gum plate. The suspended cells of strain KA5–B^T^ were inoculated on respective plates by streaking according to the long side of the filter paper and incubated at 30°C for 14 d.

The degradations of agar, starch, and dextrin were detected using Lugol staining (31). Degradations of the other polysaccharides were detected using Congo red staining (37) followed by decolorization with 1.0 M NaCl solution. Degradation abilities using both staining methods were confirmed by the appearance of a clear zone surrounding the KA5–B^T^ colony.

### Macroscopic and microscopic observations

Routine observations were performed to morphologically characterize strain KA5–B^T^. The features of the colonies were investigated after they were incubated on gellan gum plates. Gram-staining, motility, morphology, cell size, and spore formation were examined with the cells cultured on the gellan gum plate and liquid medium using a phase-contrast microscope (Olympus, Tokyo, Japan) and scanning electron microscope (SEM) (Japan Electron Optics Laboratory, Tokyo, Japan). Gram-staining was conducted after cells were stained according to Hucker’s method (27). Cultured cells that reached the death phase were also used to assess spore formation. Regarding the observation by the scanning electron microscopy, cells were washed with 10 mM phosphate buffer solution (pH 7.0) and collected on the nucleopore filter. After freeze-drying, cells were vapor-coated with carbon and examined under the high vacuum mode at 5 kV.

### Physiological analysis

The growth ranges of strain KA5–B^T^ were investigated using cells grown on the gellan gum plate or liquid culture. Temperature ranges were investigated on gellan gum plates incubated at 10°C, 15°C, 20°C, 30°C, 35°C, 37°C, and 42°C. The pH ranges and NaCl concentrations were investigated in the liquid culture. The pH was prepared from 4.0 to 10.0 at intervals of 0.5 and the NaCl concentrations used were 0%, 1%, 2%, and 3%. The optimal temperature, pH, and NaCl concentration were also investigated simultaneously. Gellan gum plates inoculated with the strain was packed in an AnaeroPouch bag (Mitsubishi Gas Chemical Company, Tokyo, Japan) and incubated under anaerobic conditions for the anaerobic growth test.

Cytochrome oxidase and catalase activities and Gram-staining were investigated using cells grown on gellan gum plates that were incubated for 5 d. Cytochrome oxidase activity was detected by the method using oxidase-testing paper (Nissui Pharmaceutical, Tokyo, Japan). Catalase activity was observed as the formation of bubbles in a 3% H_2_O_2_ solution.

### Chemotaxonomic analysis

Cells cultured in liquid medium were used to establish whole-cell fatty acid profiles, analyses of the main quinones, and the G+C content mol (%) of genomic DNA. Quinones and whole-cell fatty acids were extracted with chloroform/methanol (2:1, v/v) and purified using the method described by Hiraishi *et al.* (14). After the purification, whole-cell fatty acids and quinones were analyzed using the GC-based Microbial Identification System (MIDI, Newark NJ, USA) and Shimadzu LC–10 HPLC system (Shimadzu, Kyoto, Japan), respectively. The G+C content (mol%) of DNA was determined by HPLC (18). Bacterial DNA was extracted using the same protocol as that described for genomic DNA extraction and digested with nuclease according to the manufacturer’s instructions (Yamasa, Choshi, Japan).

### Examination of substrate oxidation

Biolog GN2 microplates (Biolog, Hayward, CA, USA) were used in the absence of culture supernatant from companions to test the oxidation of various carbon sources. KA5–B^T^ cells were cultured in liquid medium and washed with 10 mM sodium phosphate buffer (pH 7.0). Portions of cell suspensions were dispensed and incubated. Visual observations were performed after the incubation according to the manufacturer’s instructions.

### Nucleotide sequence accession number

The sequences obtained in this study were uploaded and are available on the DNA Data Banks under the accession numbers; AB300444 for KA5–A and AB174844 for KA5–B^T^.

## Results and Discussion

### Phylogenetic affiliation of strain KA5–A

To investigate the phylogenetic affiliation of strain KA5–A, which was obtained as a companion bacterium when strain KA5–B^T^ was isolated, the 16S rRNA gene of strain KA5–A was partially sequenced (corresponding to position 28–1524 of the *Escherichia coli* 16S rRNA gene), and the obtained sequence showed 99.2% and 99.0% similarities with *Mesorhizobium tamadayense* (FN563430) (23) and *Mesorhizobium tianshanense* (JN685304) (unpublished data). Based on its phylogenetic composition, the strain was regarded as a member of the genus *Mesorhizobium* (*Mesorhizobium* sp. strain KA5–A).

### Commensal interaction that enhanced growth and polysaccharide-degrading ability

The companion bacterium KA5–A is a member of the genus *Mesorhizobium*, which belongs to the order *Rhizobiales* in *Alphaproteobacteria*; therefore, we investigated the phylogenetic range needed to achieve growth enhancements in strain KA5–B^T^ with the production of metabolite(s).

**Fig. 1:**
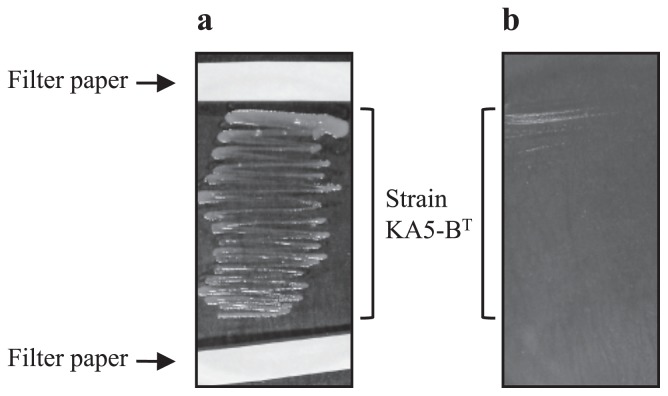
Examination of the commensal interaction that enhanced the growth of strain KA5–B^T^ by cell–free supernatants of the companions. The filter paper in Fig. 1a contained cell–free supernatants of strain KA5–A. The plate without filter paper was also prepared as a control (Fig. 1b).

As shown in Fig. 1, a growth enhancement was observed in strain KA5–B^T^ on the plate with filter papers containing the cell-free supernatants derived from strain KA5–A (Fig. 1a). However, KA5–B^T^ did not grow well without filter paper (Fig. 1b). Weak growth was only observed at locations inoculated with a high density of cells. Growth enhancements in strain KA5–B^T^ were also detected in all six bacteria tested that belonged to the order *Rhizobiales*, but were not in the two species, *Brevundimonas subvibrioides* NBRC 16000 and *Sphingomonas pruni* NBRC 15498 in *Alphaproteobacteria*, and the other genera.

The polysaccharide-degrading ability of strain KA5–B^T^ was investigated using various polysaccharides. Clear zones surrounded the KA5–B^T^ colony after the 14-d incubation in all polysaccharides tested, which were agar, arabinan, CM–cellulose, debranched arabinan, dextrin, galactomannan, larch arabinogalactan, pectic galactan, polygalacturonic acid, starch, wheat arabinoxylan, xylan, and xyloglucan.

These results indicated that the bacteria producing diffusible compounds that enhanced the growth of strain KA5–B^T^ were members of *Rhizobiales*. Tanaka *et al.* (39) isolated the novel bacterium *Catellibacterium nectariphilum* from activated sludge, and reported that this bacterium required diffusible metabolite(s) from a strain related to the genus *Sphingomonas* to enhance growth. A later study by Tanaka *et al.* (40) showed that the diffusible metabolite(s) were heat-stable, non-peptides with a low molecular weight (below 1,000 Da) produced by *Sphingomonas adhaesiva*. The KA5–B^T^ is possibly explained that growth of the strain was induced by the diffusible metabolite(s) from the bacteria of *Rhizobiales*, and the strain decomposed various polysaccharides derived from plants for anything that appeared to beneficial contribution for the bacteria of *Rhizobiales*. The identification of metabolite(s) may be important in order to more clearly elucidate bacterial interactions and establish a cultivation method for uncultivable bacteria that require metabolite(s) produced by other bacteria.

### Phylogenetic affiliation of strain KA5–B^T^ and its closest relatives

The homology search results for the almost complete 16S rRNA gene sequence (1,493 bp) of strain KA5–B^T^ showed that the taxonomic status of the obtained sequence belonged to *Gammaproteobacteria*. The most closely affiliated bacteria of strain KA5–B^T^ were Bacterium D29 (FJ654261) (unpublished data), *Steroidobacter* sp. ZUMI 137 (AB548216) (unpublished data), and *Steroidobacter denitrificans* FS^T^ (EF605262) (8) with 98.5%, 96.8%, and 96.5% similarities, respectively.

**Fig. 2:**
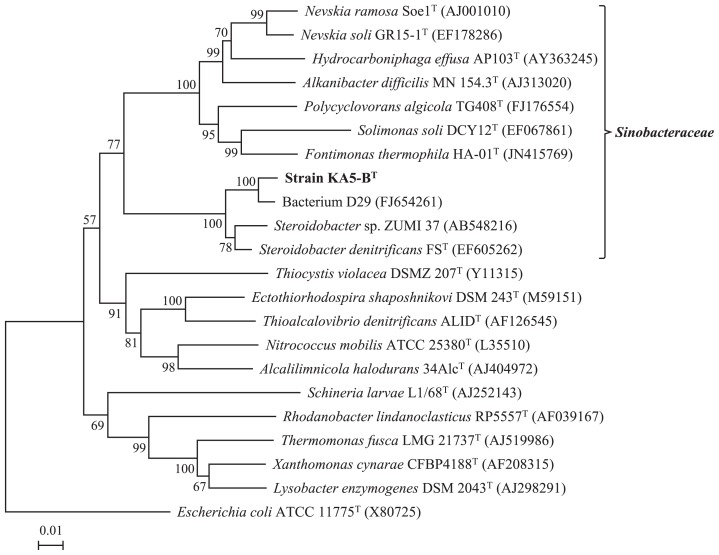
Neighbor–joining phylogenetic tree based on 16S rRNA gene sequence comparisons, showing the position of strain KA5–B^T^ and its closest relatives within the class *Gammaproteobacteria*. Accession numbers for the sequences retrieved from the databases are given in parentheses. Bootstrap percentages at each node indicate the occurrence in 1,000 bootstrapped trees. The trees based on the maximum–parsimony and maximum–likelihood methods were almost identical to that obtained with the neighbor–joining method. Bar, 0.01 substitutions per nucleotide position.

The phylogenetic analysis shown in Fig. 2 indicated that strain KA5–B^T^ belonged to the family *Sinobacteraceae* in *Gammaproteobacteria*. Genera *Alkanibacter*, *Fontimonas*, *Hydrocarboniphaga*, *Nevskia*, *Polycyclovorans* and *Solimonas*, which are members of the *Sinobacteraceae* family, were also closely related to strain KA5–B^T^. However, this strain differed from any species in these genera with the following similarities; *Alkanibacter difficilis* MN154.3^T^ (AJ313020) for 87.6%, *Fontimonas thermophila* HA–01^T^ (LN415769) for 87.2%, *Hydrocarboniphaga effusa* AP103^T^ (AY363245) for 87.4%, *Nevskia ramosa* Soe1^T^ (AJ001010) for 87.1%, *Nevskia soli* GR15–1^T^ (EF178286) for 86.9%, *Polycyclovorans algicola* TG408^T^ (FJ176554) for 86.3%, and *Solimonas soli* DCY12^T^ (EF067861) for 87.6%, which indicated that strain KA5–B^T^ belonged to a different genus to these genera.

On the other hand, strain KA5–B^T^ showed 96.5% similarity with *Steroidobacter denitrificans* FS^T^, which is the sole species in the genus *Steroidobacter*. However, this strain formed a different cluster from *Steroidobacter denitrificans* FS^T^ in the phylogenetic tree (Fig. 2), and showed the highest affiliation with Bacterium D29, which indicated that strain KA5–B^T^ is a novel species in the genus *Steroidobacter*. The similarity value of the 16S rRNA gene sequence is known to play an important role in delineating novel taxa and identifying isolates (6). Stackebrandt and Goebel (33) suggested that a 16S rRNA gene sequence similarity of 97% should become the boundary of delineation of prokaryotic species, which has been well accepted among microbiologists. Stackebrandt and Ebers (34) later proposed a more relaxed cutoff of 98.7–99%, after inspecting a large number of recently published studies. Logan *et al.* (22) and Tindall *et al.* (42) also reported that DNA-DNA hybridization, which has been employed to identify new species within a taxon, was optional for new taxa encompassing a single strain that shared more than 97% 16S rRNA gene sequence similarity to its closest neighbor. Due to these reasons, DNA-DNA hybridization was not performed between strain KA5–B^T^ and *Steroidobacter denitrificans* FS^T^ because of the similarity level of 96.5%, and the strain was regarded as a new species at the phylogenetic level.

### Morphological and physiological characteristics

**Fig. 3:**
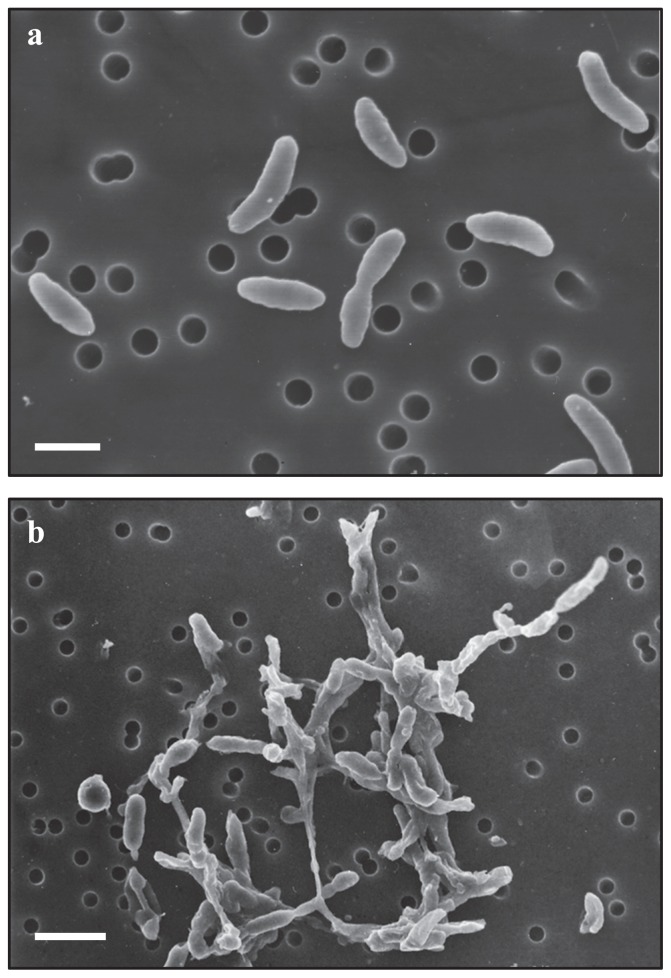
Scanning electron micrographs of strain KA5–B^T^ grown on 1.5% gellan gum plate (a) and in liquid culture (b). Bars, (a) 1 μm and (b) 2 μm.

The morphological and physiological characteristics of strain KA5–B^T^ were investigated and the results were compiled in Table 1. The strain formed smooth, circular, pale-yellow colonies on gellan gum plates. Gram-staining, motility, and cell size and shape were examined using phase-contrast microscopy and scanning electron microscopy (Fig. 3a). The cells of strain KA5–B^T^ were Gram-negative, non-motile, had a straight to slightly curved rod shape, and were 0.4–0.6 μm in width by 1.0–2.1 μm in length. These cells were included in a fibrous polysaccharide matrix when cultivated in liquid medium (Fig. 3b). Spore formation was not observed.

The physiological analysis revealed that visible colonies were detectable at 15–37°C, with optimum growth being observed at 30°C on gellan gum plates. No growth occurred at 10°C or 42°C. In addition, the strain was able to grow in liquid medium between pH 4.5 and 9.0 with optimum growth at pH 6.0–8.0. No growth was observed when 3% (w/v) NaCl was added to the liquid medium, while the strain grew at NaCl concentrations of 0%, 1%, and 2%. Metabolism was strictly aerobic and no growth occurred under anaerobic conditions. Cytochrome oxidase and catalase showed positive reactions, respectively.

### Chemotaxonomic characteristics

Whole-cell fatty acids, the main quinones, and G+C content (mol%) were analyzed after the extraction and purification of the respective targeted matter from the cells of strain KA5–B^T^. Whole-cell fatty acid profiles for KA5–B^T^ were dominated by C_16:1_ω7c (32.1%), iso–C_15:0_ (18.1%), iso–C_17:1_ω9c (11.8%), C_16:0_ (8.7%), and iso–C_17:0_ (7.9%). Other cellular fatty acids that were present at levels greater than 1% included iso–C_16:0_ (5.6%), C_12:0_ (4.7%), C_12:0_ 3–OH (4.6%), iso–C_11:0_ (3.9%), and C_12:0_ 2–OH (2.7%). The composition of respiratory quinones indicated that ubiquinone-8 (Q-8) was predominant. The G+C content of genomic DNA was 62.9 mol% (HPLC).

### Oxidation of substrates

In the oxidation test on various carbon sources in Biolog GN2 microplates, strain KA5–B^T^ oxidized the following substrates; dextrin, tween 40, L–arabinose, cellobiose, D–galactose, gentiobiose, α–D–glucose, α–D–lactose, D–melibiose, β–methyl–D–glucoside, L–raffinose, sucrose, and α–ketoglutaric acid. The oxidation of D–fructose, lactulose, maltose, L–rhamnose, D–trehalose, turanose, methyl pyruvate, DL–lactic acid, alaninamide, L–alanine, L–glutamic acid, hydroxy–L–proline, L–proline, L–threonine, and glucose 6–phosphate was weakly positive. The oxidation of the other organic substrates including acetate and citrate was negative.

### Morphological and phenotypic comparisons among strain KA5–B^T^ and its closest relatives

Table 1 shows the morphological and phenotypic characteristics of strain KA5–B^T^ and the type species of its closest relatives in the *Sinobacteraceae* family. In addition to a high sequence divergence, the closest relatives possessed different morphological and physiological characteristics. The major fatty acids were used as the predominant feature to distinguish species from each other. Compared to *Steroidobacter denitrificans* FS^T^, strain KA5–B^T^ was non-motile, strictly aerobic, and it grew on agar plates at 15°C and pH 5.0. The utilization of lactose, galactose, D–glucose, and sucrose was positive and glutamate was weakly positive, while acetate and propionate were negative. In contrast, *Steroidobacter denitrificans* FS^T^ was motile, grew anaerobically, and did not grow on agar plates at 15°C and pH 5.0. The utilization of acetate, glutamate, and propionate were positive, while lactose, galactose, D–glucose, and sucrose were negative.

Thus, strain KA5–B^T^ showed distinguishable features from *Steroidobacter denitrificans* FS^T^ at the morphological and physiological levels, which indicated that KA5–B is a novel species in the genus *Steroidobacter*. Based on the above results, we propose that strain KA5–B^T^ belongs to a novel species, for which the name *Steroidobacter agariperforans* sp. nov. is proposed.

### Description of *Steroidobacter agariperforans* sp. nov

*Steroidobacter agariperforans* (a.ga.ri.per.fo’rans. Malayan n. *agar* agar; N.L. n. *agarum* agar [algal polysaccharide]; L. part. adj. *perforans* perforating [making holes]; N.L. part. adj. *agariperforans* making holes in agar, bacterium making deep hollows in agar).

It is Gram-negative, strictly aerobic, non-motile, oxidase and catalase positive, and does not form spores. Cells have a straight to slightly curved rod shape (0.4–0.6 μm in width by 1.0–2.1 μm in length) and hydrolyze agar. Included in the fibrous polysaccharide matrix when cultivated in liquid medium. Diffusible metabolite(s) produced by bacteria in the order *Rhizobiales* may be required for vigorous growth. Colonies are circular and pale yellow in color. The temperature and pH ranges for growth are 15–37°C and between pH 4.5 and 9.0, with optimum growth occurring at 30°C and pH 6.0–8.0. No growth occurred at 10 or 42°C and NaCl concentrations more than 3% (w/v). The main cellular fatty acids are C_16:1_ω7c (32.1%), iso–C_15:0_ (18.1%), and iso–C_17:1_ω9c (11.8%). Q–8 is the main component of the quinone system. The DNA G+C content of the type strain of the type species is 62.9 mol% (by HPLC). 16S rRNA gene sequence analysis places the genus in the class *Gammaproteobacteria*. According to Biolog GN2 tests, the type strain is positive for the oxidation of dextrin, tween 40, L–arabinose, cellobiose, D–galactose, gentiobiose, α–D–glucose, α–D–lactose, D–melibiose, β–methyl–D–glucoside, L–raffinose, sucrose and α–ketoglutaric acid. The oxidation of D–fructose, lactulose, maltose, L–rhamnose, D–trehalose, turanose, methyl pyruvate, DL–lactic acid, alaninamide, L–alanine, L–glutamic acid, hydroxy–L–proline, L–proline, L–threonine, and glucose 6–phosphate was weakly positive. The oxidation of the other organic substrates including acetate and citrate in Biolog GN2 microplates was negative. The type strain is *Steroidobacter agariperforans* KA5–B^T^ (JCM 18477^T^ = KCTC 32107^T^), which was isolated from a soil sample.

## Figures and Tables

**Table 1:** Phylogenetic characteristics of strain KA5–B^T^ in comparison with its closest relatives. The data of its closest relatives were compiled from Fahrbach *et al.* (8), Friedrich and Lipski (12), Losey *et al.* (24), Palleroni *et al.* (29), Sturmeyer *et al.* (36), Gutierrez *et al.* (13), Kim *et al.* (19) for *Steroidobacter denitrificans*, *Alkanibacter difficilis*, *Fontimonas thermophilia*, *Hydrocarboniphaga effusa*, *Nevskia ramosa*, *Polycyclovorans algicola* and *Solimonas soli*, respectively. The data of Weon *et al.* (44), Kim *et al.* (20), Babenzien and Cypionka (2), and Cypionka *et al.* (7) were also used as related references. All bacteria were Gram-negative and non spore forming. Sum3 and 8 were composed of iso C_15:0_ 2 OH, C_16:1_ω7c and C_18:1_ω9c, and C_18:1_ω9c, C_18:1_ω7c, and C_18:1_ω6c, respectively. The symbols, +, − and ±, indicate positive, negative, and weakly positive, respectively. ND indicates “not determined” or “no data available”.

	Strain KA5-B^T^	*Steroidobacter denitrificans*	*Alkanibacter difficilis*	*Fontimonas thermophila*	*Hydrocarboniphaga effusa*	*Nevskia ramosa*	*Polycyclovorans algicola*	*Solimonas soli*
Similarity (%) to the 16S rRNA gene		96.5	87.6	87.2	87.4	87.1	86.5	87.6
Cell size width by length (μm)	0.4 0.6 by 1.0 2.1	0.3 0.5 by 0.6 1.6	0.5 0.7 by 0.6 1.2	0.5 0.75 by 1.0 2.0	0.75 0.85 by 1.5 2.0	0.7 1.1 by 1.5 2.3	0.5 by 1.0 1.2	0.2 0.4 by 0.3 0.5
Cell morphology	straight to slightly curved rod	slightly curved rod	small rod	rod	rod	slightly curved rod	rod	rod
Motility	non-motile	motile by single polar flagellum	ND	motile by single polar flagellum	motile by single flagellum of polar, subpolar or insertion	motile by single polar flagellum	motile by single polar flagellum	non-motile
G+C content (mol%)	62.9	61.9	62.8	64.4	60 61	67.8	64.3	40.5
Catalase	+	+	+	+	+	±	+	+
Oxydase	+	+	+	+	+	+	+	+
Main quinone	Q-8	Q-8	Q-8	Q-8	ND	ND	Q-8	Q-8
Main fatty acids (≥10%)	iso-C_15:0_, C_16:1_ω7c, iso-C_17:1_ω9c	C_15:0_, C_17:1_ω8c	C_16:0_, C_18:0_, C_18:1_cis11, C_19:0_cyclo11–12	Iso-C_16:0_, C_16:1_ω5c, sum8	C_12:0_, C_16:0_, C_16:1_cis9, C_18:1_cis11	C_14:0_, C_18:1_ω7c, sum3	C_16:0_, C_16:1_ω7c, C_18:1_ω7c	C_16:0_, C_18:0_, sum3
Growth on an agar plate	+	−	+	+	+	+	+	+
Anaerobic growth	−	+	−	−	−	−	−	+
Growth at 15°C	+	−	−	−	+	+	+	−
Growth at pH 5.0	+	−	ND	−	ND	ND	−	−
Utilization of:								
Acetate	−	+	−	−	±/−	+	+	+
Citrate	−	−	−	−	ND	ND	ND	−
Galactose	+	−	−	−	−	ND	ND	ND
D-Glucose	+	−	−	−	−	+	ND	+
Glutamate	±	+	−	±/−	+	+	±	ND
Lactose	+	−	−	−	+	+	ND	−
Propionate	−	+	−	−	+	−	+	±
Sucrose	+	−	−	−	+/±	+/−	ND	−

## References

[1] Agbo JAC, Moss MO (1979). The isolation and characterization of agarolytic bacteria from a lowland river. J Gen Microbiol.

[2] Babenzien HD, Cypionka H, Garrity GM, Brenner DJ, Krieg NR, Staley J (2005). Genus V. *Nevskia* Famintzin 1892, 484^AL^. Bergey’s Manual of Systematic Bacteriology, the *Proteobacteria*, Part B the *Gammaproteobacteria*.

[3] Bacic A, Moody SF, Clarke AE (1986). Structural analysis of secreted root slime from maize. Plant Physiol.

[4] Bannikova GE, Lopatin SA, Varlamov VP, Kuznetsov BB, Kozina IV, Miroshnichenko ML, Chernykh NA, Turova TP, Bonch-Osmolovskaya EA (2008). The thermophilic bacteria hydrolyzing agar: characterization of thermostable agarase. Appl Biochem Microbiol.

[5] Chaboud A, Rougier M (1984). Identification and localization of sugar components of rice (*Oryza sativa* L.) root cap mucilage. J Plant Physiol.

[6] Chun J, Lee JH, Jung Y, Kim M, Kim S, Kim BK, Lim YW (2007). ExTaxon: a web-based tool for the identification of prokaryotes based on 16S ribosomal RNA gene sequences. Int J Syst Bacteriol.

[7] Cypionka H, Babenzien HD, Glockner FO, Amann R, Dworkin M, Falkow S, Rosenberg E, Schleifer KH, Stackebrabdt E (2006). The Genus *Nevskia*. The prokaryotes, A Hand Book on the Biology of Bacteria: *Proteobacteria*: Gamma Subclass.

[8] Fahrbach M, Kuever J, Remesch M, Huber BE, Kämpfer P, Dott W, Hollender J (2008). *Steroidobacter denitrificans* gen. nov., sp. nov., a steroidal hormone–degrading gammaproteobacterium. Int J Syst Evol Microbiol.

[9] Felsenstein J (1981). Evolutionary trees from DNA sequences: a maximum likelihood approach. J Mol Evol.

[10] Felsenstein J (1985). Confidence limits on phylogenies: an approach using the bootstrap. Evolution.

[11] Fitch WM (1971). Toward defining the course of evolution: minimum change for a specific tree topology. Syst Zool.

[12] Friedrich MM, Lipski A (2008). *Alkanibacter difficilis* gen. nov., sp. nov. and *Singularimonas variicoloris* gen. nov., sp. nov., hexane–degrading bacteria isolated from a hexane–treated biofilter. Int J Syst Evol Microbiol.

[13] Gutierrez T, Green DH, Nichols PD, Whitman WB, Semple KT, Aitken MD (2013). *Polycyclovorans algicola* gen nov., sp. nov., an aromatic-hydrocarbon-degrading marine bacterium found associated with laboratory cultures of marine phytoplankton. Appl Environ Microbiol.

[14] Hiraishi A, Muramatsu K, Ueda Y (1996). Molecular genetic analyses of *Rhodobacter azotoformans* sp. nov. and related species of phototrophic bacteria. Syst Appl Microbiol.

[15] Hosoda A, Sakai M (2006). Isolation of *Asticcacaulis* sp. SA7, a novel agar–degrading Alphaproteobacterium. Biosci Biotechnol Biochem.

[16] Hosoda A, Sakai M, Kanazawa S (2003). Isolation and characterization of agar–degrading *Paenibacillus* spp. associated with the rhizosphere of spinach. Biosci Biotechnol Biochem.

[17] Hunger W, Claus D (1978). Reisolation and growth conditions of *Bacillus* agar-exedens. Antonie van Leeuwenhoek.

[18] Katayama-Fujimura Y, Komatsu Y, Kuraishi H, Kaneko T (1984). Estimation of DNA base composition by high performance liquid chromatography of its nuclease P1 hydrolysate. Agric Biol Chem.

[19] Kim MK, Kim YJ, Cho DH, Yi TH, Soung NK, Yang SC (2007). *Solimonas soli* gen nov., sp. nov., isolated from soil of a ginseng field. Int J Syst Evol Microbiol.

[20] Kim SJ, Weon HY, Kim YS, Park IC, Son JA, Kwon SW (2011). *Nevskia terrae* sp. nov., isolated from soil. Int J Syst Evol Microbiol.

[21] Knee EM, Gong FC, Gao M, Teplitski M, Jones AR, Foxworthy A, Mort AJ, Bauer WD (2001). Root mucilage from pea and its utilization by rhizosphere bacteria as a sole carbon source. Mol Plant Microbe Interact.

[22] Logan NA, Berge O, Bishop AJ (2009). Proposal minimal standards for describing new taxa of aerobic, endospore–forming bacteria. Int J Syst Evol Microbiol.

[23] Lorite MJ, Donate-Correa J, del Arco-Aguilar M, Perez GR, Sanjuan J, Leon-Barrios M (2010). Lotus endemic to the Canary Islands are nodulated by diverse and novel rhizobial species and symbiotypes. Syst Appl Microbiol.

[24] Losey NA, Stevenson BS, Verbarg S, Rudd S, Moore ERB, Lawson PA (2013). *Fontimonas thermophilia,* gen. nov., sp. nov., a moderately thermophilic bacterium isolated from a freshwater hot spring. Proposal of *Solimonadaceae* fam. nov. to replace *Sinobacteraceae* Zhou *et al.* 2008. Int J Syst Evol Microbiol.

[25] Miyazaki M, Nogi Y, Ohta Y, Hatada Y, Fujiwara Y, Ito S, Horikoshi K (2008). *Microbulbifer agarilyticus* sp. nov. and *Microbulbifer thermotolerans* sp. nov., agar–degrading bacteria isolated from deep–sea sediment. Int J Syst Evol Microbiol.

[26] Moody SF, Clarke AE, Bacic A (1988). Structural analysis of secreted slime from wheat and cowpea roots. Phytochemistry.

[27] Murray RGE Doetsch RN Robinow CF 1994Determinative and cytological light microscopy32 Gerhardt P Murray RGE Wood WA Krieg NR Methods for General and Molecular Bacteriology American Society for Microbiology Washington, D.C

[28] Ohta Y, Nogi Y, Miyazaki M, Li Z, Hatada Y, Ito S, Horikoshi K (2004). Enzymatic properties and nucleotide and amino acid sequences of a thermostable β–agarase from the novel marine isolate, JAMB–A95. Biosci Biotechnol Biochem.

[29] Palleroni NJ, Port AM, Chang HK, Zylstra GJ (2004). *Hydrocarboniphaga effusa* gen. nov., sp. nov., a novel member of the γ–*Proteobacteria* active in alkane and aromatic hydrocarbon degradation. Int J Syst Evol Microbiol.

[30] Pitcher DG, Saunders NA, Owen RJ (1989). Rapid extraction of bacterial genomic DNA with guanidium thiocyanate. Lett Appl Microbiol.

[31] Ruijssenaars HJ, Hartmans S (2001). Plate screening methods for the detection of polysaccharase–producing microorganisms. Appl Microbiol Biotechnol.

[32] Saitou N, Nei M (1987). The neighbor-joining method: a new method for reconstructing phylogenetic trees. Mol Biol Evol.

[33] Stackebrandt E, Goebel BM (1994). Taxonomic note: a place for DNA-DNA reassociation and 16S rRNA sequence analysis in the present species definition in bacteriology. Int J Syst Bacteriol.

[34] Stackebrandt E, Ebers J (2006). Taxonomic parameters revisited: tarnished gold standards. Microbiol Today.

[35] Stanier RY (1942). Agar-decomposing strains of the *Actinomyces coelicolor* species–group. J Bacteriol.

[36] Stürmeyer H, Overmann J, Babenzien HD, Cypionka H (1998). Ecophysiological and phylogenetic studies of *Nevskia ramose* in pure culture. Appl Environ Microbiol.

[37] Suyama K, Yamamoto H, Naganawa T, Iwata T, Komada H (1993). A plate count method for aerobic cellulose decomposers in soil by congo red staining. Soil Sci Plant Nutr.

[38] Tamura K, Peterson D, Peterson N, Stecher G, Nei M, Kumar S (2011). MEGA5: molecular evolutionary genetics analysis using maximum likelihood, evolutionary distance, and maximum, parsimony methods. Mol Biol Evol.

[39] Tanaka Y, Hanada S, Manome A, Tsuchida T, Kureane R, Nakamura K, Kamagata Y (2004). *Catellibacterium nectariphilum* gen. nov., sp. nov., which requires a diffusible compound from a strain related to the genus *Sphingomonas* for vigorous growth. Int J Syst Evol Microbiol.

[40] Tanaka Y, Hanada S, Tamaki H, Nakamura K, Kamagata Y (2005). Isolation and identification of bacterial strains producing diffusible growth factor(s) for *Catellibacterium nectariphilum* strain AST4^T^. Microbes Environ.

[41] Thompson JD, Higgins DG, Gibson TJ (1994). CLUSTAL W: improving the sensitivity of progressive multiple sequence alignment through sequence weighting, position–specific gap penalties and weight matrix choice. Nucleic Acids Res.

[42] Tindall BJ, Rosselló–Móra R, Busse HJ, Ludwig W, Kämpfer P (2010). Notes on the characterization of prokaryote strains for taxonomic purposes. Int J Syst Evol Microbiol.

[43] van der Meulen HJ, Harder W, Veldkamp H (1974). Isolation and characterization of *Cytophaga flevensis* sp. nov., a new agarolytic flexibacterium. Antonie van Leeuwenhoek.

[44] Weon HY, Kim BY, Son JA, Song MH, Kwon SW, Go SJ, Stackebrandt E (2008). *Nevskia soli* sp. nov., isolated from soil cultivated with Korean ginseng. Int J Syst Evol Microbiol.

[45] Winker S, Woese CR (1991). A definition of the domains Archaea, Bacteria and Eucarya in terms of small subunit ribosomal RNA characteristics. Syst Appl Microbiol.

[46] Zhilina TN, Zavarzin GA, Rainey F, Kevbrin VV, Kostrikina NA, Lysenko AM (1996). *Spirochaeta alkalica* sp. nov., *Spirochaeta africana* sp. nov., and *Spirochaeta asiatica* sp. nov., alkaliphilic anaerobes from the continental soda lakes in central Asia and the east African rift. Int J Syst Evol Microbiol.

